# Thrombin Enhances NGF-Mediated Neurite Extension via Increased and Sustained Activation of p44/42 MAPK and p38 MAPK

**DOI:** 10.1371/journal.pone.0103530

**Published:** 2014-07-25

**Authors:** Rania E. Mufti, Krishna Sarker, Yan Jin, Songbin Fu, Jesusa L. Rosales, Ki-Young Lee

**Affiliations:** 1 Departments of Cell Biology and Anatomy, Southern Alberta Cancer Research and Hotchkiss Brain Institutes, University of Calgary, Calgary, Alberta, Canada; 2 Biochemistry and Molecular Biology, Snyder Institute for Chronic Diseases, University of Calgary, Calgary, Alberta, Canada; 3 Laboratory of Medical Genetics, Harbin Medical University, Harbin, China; Chang Gung University, Taiwan

## Abstract

Rapid neurite remodeling is fundamental to nervous system development and plasticity. It involves neurite extension that is regulated by NGF through PI3K/AKT, p44/42 MAPK and p38 MAPK. It also involves neurite retraction that is regulated by the serine protease, thrombin. However, the intracellular signaling pathway by which thrombin causes neurite retraction is unknown. Using the PC12 neuronal cell model, we demonstrate that thrombin utilizes the PI3K/AKT pathway for neurite retraction in NGF-differentiated cells. Interestingly, however, we found that thrombin enhances NGF-induced neurite extension in differentiating cells. This is achieved through increased and sustained activation of p44/42 MAPK and p38 MAPK. Thus, thrombin elicits opposing effects in differentiated and differentiating cells through activation of distinct signaling pathways: neurite retraction in differentiated cells via PI3K/AKT, and neurite extension in differentiating cells via p44/42 MAPK and p38 MAPK. These findings, which also point to a novel cooperative role between thrombin and NGF, have significant implications in the development of the nervous system and the disease processes that afflicts it as well as in the potential of combined thrombin and NGF therapy for impaired learning and memory, and spinal cord injury which all require neurite extension and remodeling.

## Introduction

Neurite remodeling, which involves rapid extension and retraction, is crucial for nervous system development and plasticity [1]. It requires neurotrophins such as nerve growth factor (NGF) for neurite extension during brain development [2]. NGF binds to TrkA/p75*^NTR^* receptor tyrosine kinases and their interaction causes receptor dimerization and autophosphorylation at their tyrosine residues. This results in the formation of docking sites for SH2 domain containing proteins which allows coupling of TrkA/p75*^NTR^* receptors to intracellular signaling cascades [3]. In rat pheochromocytoma PC12 cells, a commonly used model for sympathetic neurons [4], treatment with NGF induces neurite outgrowth in serum-starved condition via the activation of the mitogen-activated protein kinase/extracellular regulated kinase (MAPK/ERK) pathways that include p44/42 MAPK and p38 MAPK [5]. In addition, NGF exerts a neuroprotective effect on PC12 cells by activating the phosphatidylinositol-3 kinase (PI3K)/AKT pathway which is important for cell survival [6]. AKT is involved in the modulation of the mitochondrial membrane potential [7] and phosphorylation of AKT downstream targets results in the blockade of apoptosis [8].

Prothrombin and its active form, thrombin, a serine protease, have been detected in the developing and adult rat brains [9], [10], [11], [12], [13]. Prothrombin is detectable as early as embryonic day 13 in the developing rat brain followed by a biphasic regulation as prothrombin level decreases up until birth and subsequently increase until adulthood [10]. Prothrombin transcripts are found throughout the neonatal and adult rat nervous system, including the olfactory bulb, cortex, thalamus, hypothalamus, basal ganglia, hippocampus, and cerebellum [10]. During brain development, thrombin has been implicated to have neuromodulatory roles, including neurite retraction [14], [15], [16]. It has been shown to activate a G protein-coupled receptor 1 (proteinase-activated receptor-1; PAR-1) [12], [15], [17] by cleaving its extracellular N-terminal domain between Arg42 and Ser43. This cleavage exposes a new N-terminus that functions as a tethered ligand (SFLLRN) and that interacts distally within PAR-1 to activate G protein-coupled signal transduction pathways [18]. A synthetic peptide containing the tethered ligand sequence, SFLLRN, was also shown to activate PAR-1 receptor [17]. Thrombin activation of PAR-1 has been shown to induce growth cone collapse and neurite retraction that requires cytoskeletal rearrangement [15], [16], [19], [20]. The retraction of neurites and subsequent cell rounding is inhibited by kinase inhibitors [19], indicating the involvement of PAR-1 receptor signaling. In response to peripheral nervous system (PNS) injury, thrombin and its inhibitors are both synthesized locally by Schwann cells [21]. It is possible that interplay among thrombin, its inhibitors and neurotrophic factors is important for PNS regeneration and potentially proper nervous system development. Interestingly, thrombin is a mitogen that induces proliferation of a variety of smooth muscle types [22], [23], [24]. This is accomplished by activation of PAR-1 followed by p44/p42 MAPK and p38 MAPK [22], [23], [24]. Inhibitors of MEK1/2 and p38 MAPK attenuate the response, supporting its mitogenic role [22], [23], [24]. In addition, thrombin has been shown to induce IL-8 release through PAR-1 mediated p44/p42 and p38 MAPK signaling pathways in dermal fibroblasts [25]. Interestingly, thrombin-mediated signaling pathways leading to cell proliferation and IL-8 secretion in dermal fibroblasts are similar to those induced by NGF in PC12 cells.

As NGF and thrombin coexist during nervous system development and remodeling, and activate signaling pathways that cause opposite effects in neurons, we sought to further characterize their opposing signaling effects on neurite extension in differentiating and differentiated cells. Interestingly, we found that although thrombin induces neurite retraction in differentiated PC12 cells through the AKT pathway, we unexpectedly determined that thrombin and NGF act synergistically on differentiating cells to increase neurite extension. The latter is achieved through enhanced phosphorylation of p44/p42 and p38 MAPKs. Our findings suggest a novel cooperative role between thrombin and NGF during nervous system development and remodeling.

## Materials and Methods

### Materials

NGF and thrombin were purchased from Sigma. Kinase inhibitors (U0126, LY294002 and SB203580) were purchased from Calbiochem. Antibodies for p38 MAPK, phospho-p38 MAPK (Thr180/Tyr182), p44/p42 MAPK, phospho-p44/p42 MAPK (Thr202/Tyr204), AKT, and phospho-AKT (Ser473) were purchased from Cell Signaling. Actin (I-19) antibody was purchased from Santa Cruz Biotech.

### Cell culture

PC12 cells (ATCC CRL-1721) derived from a pheochromocytoma of a rat adrenal medulla, and commonly used as a model system for sympathetic neurons, were cultured in 10-cm dishes in serum-rich proliferating media (PM: RPMI 1640 with L-glutamine, 5% fetal bovine serum, 10% horse serum, 100 units penicillin and 100 mg streptomycin) at 37°C with 5% CO_2_. Cells were passaged three times per week using 0.25% trypsin-EDTA solution (Invitrogen).

### SDS-PAGE and western blot analysis

Cells were lysed in 40 µl of boiling 1× sample buffer (62.5 mM Tris-HCl pH 6.8, 12.5% glycerol, 1.25% SDS, and 1.25% β-mercaptoethanol). Prior to SDS-PAGE analysis, lysates were centrifuged at 15,000 rpm for 30 sec, sonicated and boiled for 5 min at 95°C. Cell lysates were subjected to SDS-PAGE (12.5%) and transferred to polyvinylidene fluoride (PVDF) membrane (Millipore) in transfer buffer (25 mM Tris base, 200 mM glycine, and 20% (v/v) methanol, pH = 8.3). After blocking in 5% non-fat milk in Tris-buffered saline with Tween-20 (TBS/T: 10 mM Tris-HCl, pH 8.0, 150 mM NaCl, 2.6 mM KCl, 0.1% Tween-20) for 1 hr at room temperature, membranes were probed with the appropriate primary antibody. After 4 washes with TBS/T, membranes were incubated with the appropriate horseradish peroxidase-conjugated secondary antibody (1∶2500; incubated for 1 hr at room temperature). The ECL western blotting detection reagents (GE Healthcare) were used to detect the presence of immunoreactive bands. In all experiments, TBS/T was used for antibody dilution.

### Differentiation and neurite retraction assays and time-lapse microscopy

PC12 cells plated in serum-rich PM at a density of 1.5×10^5^ cells per 35-mm plate were switched to differentiating media (DM; RPMI 1640 with L-glutamine, 0.2% horse serum, and 100 unit penicillin/100 mg streptomycin) after 24 hrs. After serum starvation (in DM) for 18 hours, cells were treated with NGF (100 ng/ml) to induce differentiation. Differentiated PC12 cells were then photographed after treatment as indicated (48 or 72 hours) using an Olympus IX71 inverted microscope. For neurite retraction assay, differentiated PC12 cells were exposed to thrombin (1 unit/ml) and retraction of neurites was monitored and photographed every minute for 1 hour using an Olympus 1X17 inverted microscope. Live cell images and length of neurites of cells treated with NGF or NGF+thrombin were analyzed using the Image-Pro software (Olympus).

### Statistical analysis

All statistical analysis was done using one-way ANOVA in SPSS 13.0. Data was considered significant only when the p-value was smaller than 0.05.

## Results and Discussion

### Thrombin induces neurite retraction in differentiated cells via activation of AKT but not p44/42 and p38 MAPK

**Figure 1:**
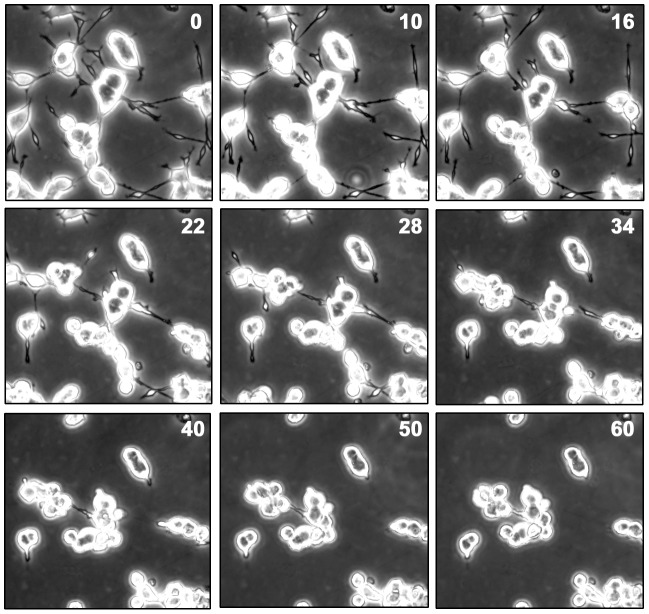
Thrombin causes neurite retraction in differentiated PC12 cells. PC12 cells were seeded and cultured as described in Materials and Methods. After treatment with NGF (100 ng/ml) for 72 hours to induce differentiation, cells were exposed to thrombin (I unit/ml). Cells were then analyzed by time-lapse microscopy (Olympus 1X17). The numbers on the top right corner of every panel indicate the time (min) after addition of thrombin. The experiment was repeated four times.

Thrombin is known to stimulate neurite retraction but the intracellular signaling pathway by which thrombin induces neurite retraction remains to be investigated. To address this issue, we used PC12 cells that were induced to differentiate by treatment with NGF for 72 hours. Differentiated cells were then exposed to thrombin and examined by time-lapse microscopy. As shown in Figure 1, NGF-differentiated cells (time 0) generally exhibited long neurites. After 22 min in the presence of thrombin (1 u/ml), there was clear retraction of a significant number of neurites and by 60 min, most of the neurites (95%) have disappeared, demonstrating a dramatic retraction of PC12 cell neurites resulting from exposure to thrombin. It is worth noting that thrombin had a dose-response effect. We observed that lower concentrations (e.g. 0.1 u/ml) caused lesser and slower retraction while higher concentrations (e.g. 2 to 3 u/ml) caused a more rapid retraction (data not shown). However, for our time-lapse microscopy, 1 u/ml allowed us to optimally observe thrombin-induced neurite retraction, noting decreasing neurite length approximately every 10 seconds as shown in Fig. 1. For consistency, we have used 1 u/ml for our subsequent experiments.

**Figure 2:**
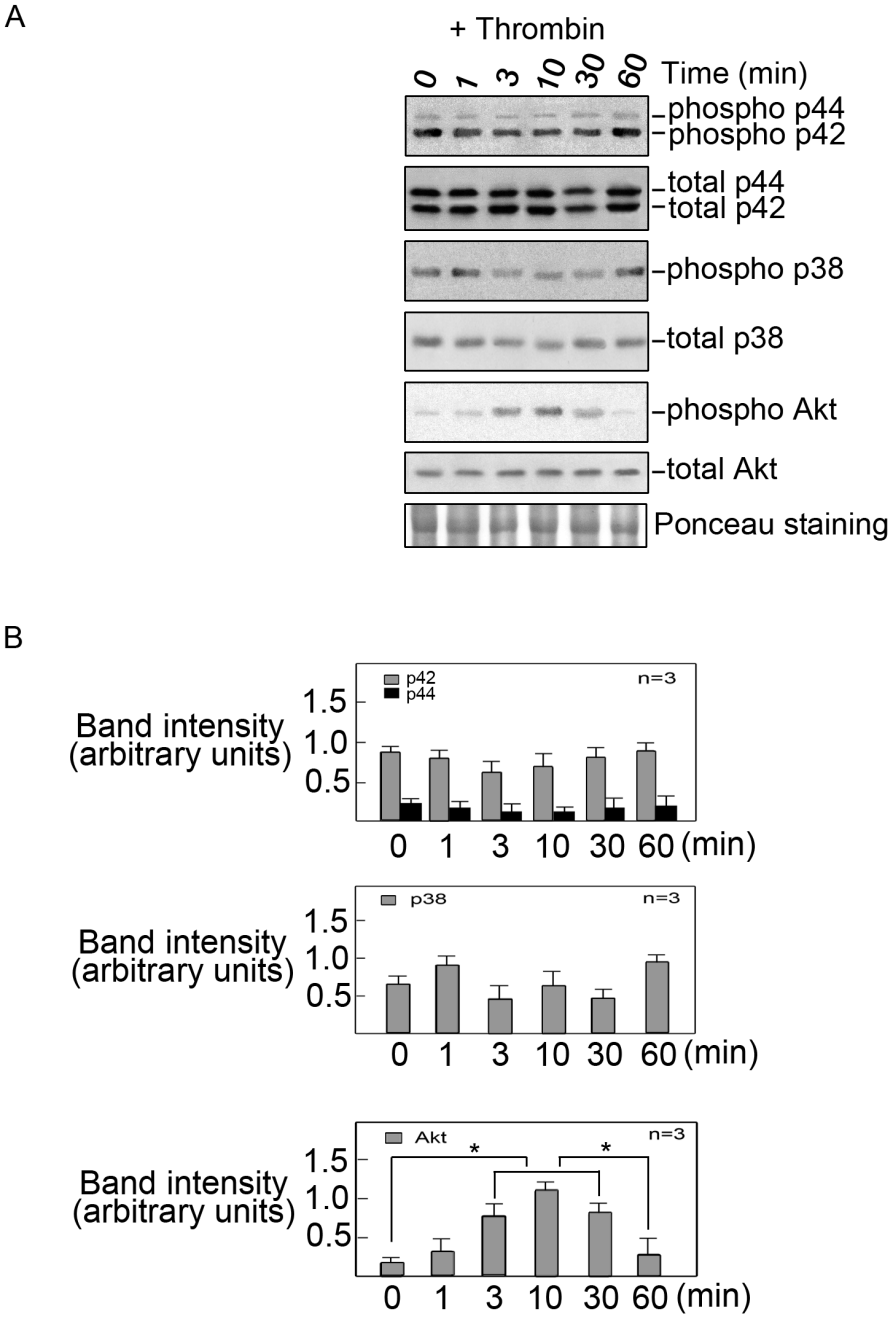
AKT is activated during thrombin-induced neurite retraction in differentiated PC12 cells. A. NGF-differentiated PC12 cells (treated with NGF for 72 hrs) were treated with thrombin (1 unit/ml) to induce neurite retraction. Cells were lysed at various time points (0, 1, 3, 10, 30 and 60 min) following thrombin treatment and lysates were subjected to western blot analysis using phospho-antibodies to p44/42 MAPK, p38 MAPK, and AKT. Immunoblots for total amounts of these proteins were used as internal controls. Equivalent protein loading of samples was further assessed by Ponceau staining. B. Intensities of the immunoreactive bands (in arbitrary units) in (A) and two other representative blots were determined by densitometric analysis using the NIH Image 1.61 software. Values are means±SD of the three representative blots that were analyzed by densitometric scanning. Students t-test was used to analyze data with p<0.05 considered significant and indicated by an asterisk (*).

Previously, thrombin has been shown to induce proliferation of a variety of smooth muscle types through activation of PAR-1 followed by p44/p42 MAPK and p38 MAPK activation [22], [23], [24]. In addition, thrombin has been shown to stimulate both growth and contractibility of dermal papilla cells through activation of the PI3K/Akt pathway [26]. Thus, we sought to determine whether p38 MAPK, p44/42 MAPK and AKT are involved in the thrombin-induced neurite retraction in PC12 cells. NGF-differentiated cells were treated with thrombin at various time points and activation of p44/42 MAPK, p38 MAPK and AKT were evaluated. By western blot analysis using phospho-specific antibodies, we found that p44/42 MAPK and p38 MAPK had only modest changes in activity following thrombin treatment (Figure 2A and Figure 2B, top panels). However, we found that AKT was clearly activated 3 min after thrombin treatment (Figure 2A 3^rd^ panel, lane 3; Figure 2B bottom panel). AKT activation reached a peak at 10 min and declined at 30 min following thrombin treatment. Thus, it appears that thrombin induces neurite retraction in NGF-differentiated PC12 cells through the activation of AKT but not p44/42 and p38 MAPK. However, we found that inhibition of AKT only partially antagonized thrombin-induced neurite retraction (data not shown), pointing to the possibility that another factor may be involved in thrombin-induced neurite retraction. Further investigation is required to test this possibility but nonetheless, our observations provide strong evidence that AKT plays an important role in thrombin-induced neurite retraction in differentiated PC12 cells.

### Thrombin synergistically enhances NGF-induced neurite outgrowth in differentiating PC12 cells

**Figure 3:**
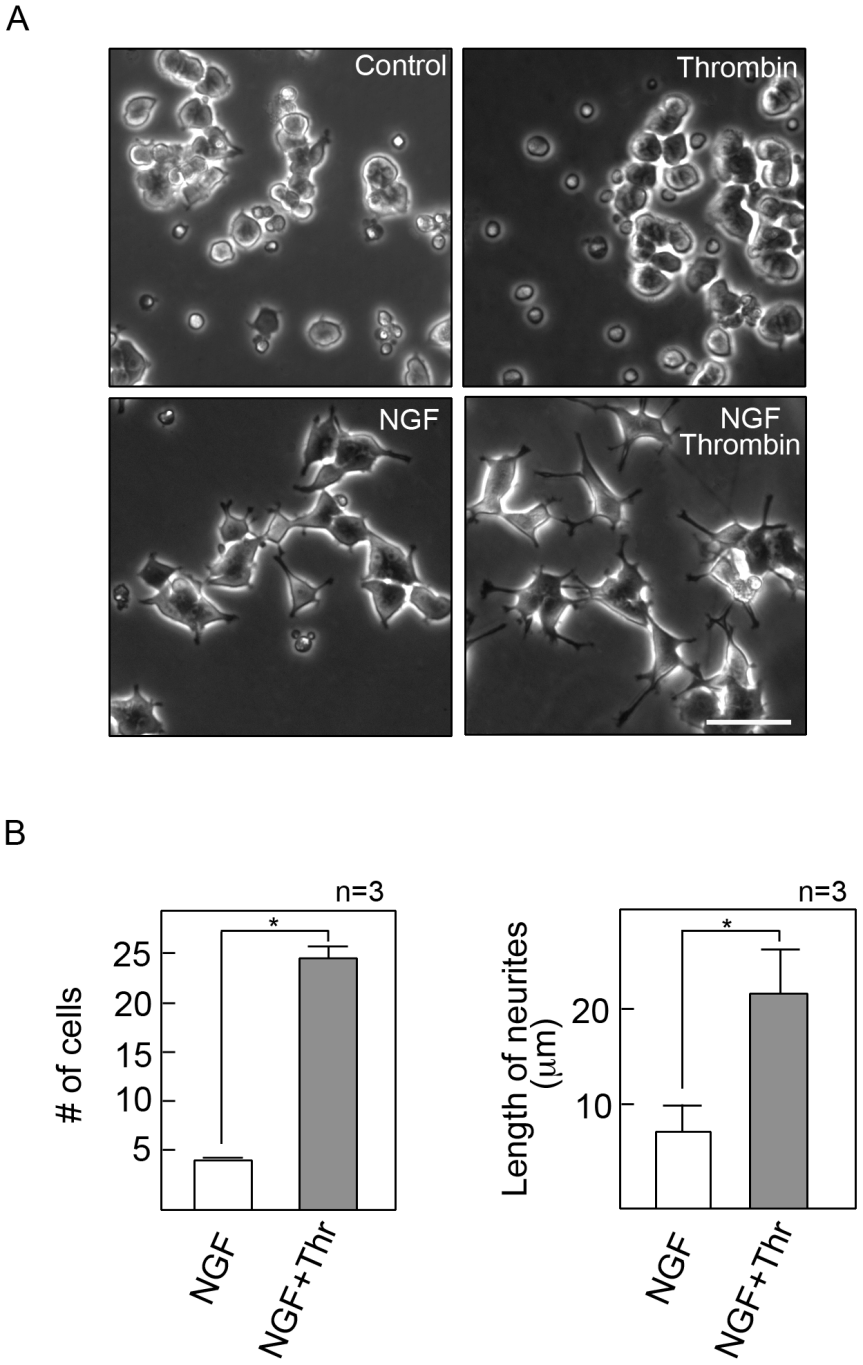
Thrombin synergistically enhances NGF-induced neurite outgrowth in differentiating PC12 cells. A. PC12 cells were plated as described in Materials and Methods. After 18 hours of serum starvation in DM, cells were treated with thrombin alone (upper right panel), NGF alone (lower left panel), or NGF and thrombin simultaneously (lower right panel) under serum-starved conditions. The upper left panel shows cells that were not treated with either thrombin or NGF. Photographs were taken 48 hours after treatment. Scale bar = 60 µm. B. The left panel shows the number of cells with neurites longer than 20 µm in the NGF and NGF+thrombin treatment groups. The right panel compares the average length of neurites in the NGF and NGF+thrombin treatment groups. Measurements were taken from 3 sets of 150 cells for each treatment group. The three sets were from 3 representative experiments. Statistical significance, using one-way ANOVA, was set at p≤0.01 and indicated by an asterisk (*). Nerve growth factor, NGF; Thrombin, Thr.

Our observation that thrombin retracts neurites in NGF-differentiated cells led us to examine whether thrombin would inhibit growth of neurites in cells that are being induced to differentiate using NGF. In other words, we sought to examine whether thrombin would inhibit the growth of neurites when added simultaneously with NGF. As shown in Figure 3A, thrombin by itself did not cause a significant change in overall cell morphology although it appeared to promote some cell death (upper right panel) compared to untreated control cells (upper left panel). As expected, NGF induced neurite outgrowth in PC12 cells (lower left panel). Surprisingly, however, simultaneous treatment with NGF and thrombin resulted in longer neurites (lower right panel). Further analysis of cells treated with NGF alone and NGF+thrombin (Figure 3B) revealed a significant increase in number of cells with long (≥20 µm) neurites (left panel) and in length of neurites (right panel) in NGF+thrombin-treated cells. Thus, while thrombin causes neurite retraction in NGF-differentiated cells, thrombin enhances neurite extension in cells that are differentiating in response to NGF. The latter indicates a novel synergistic effect of NGF and thrombin as well as a novel mechanism for neurite extension that have not been previously addressed.

### Thrombin augments NGF-induced neurite outgrowth in differentiating cells via enhanced and sustained activation of p44/42 and p38 MAPK

The opposing effects of thrombin in differentiated and differentiating PC12 cell neurites suggest stimulation of distinct signaling pathways in these cells. We have found that AKT activation is involved in thrombin retraction of neurites in NGF-differentiated cells and thus, our next step was to determine the signaling pathways involved in thrombin enhancement of NGF-induced neurite extension. As mentioned above, studies in the past have demonstrated that NGF promotes differentiation and neurite extension via activation of p44/p42 MAPK and p38 MAPK [27], [28], [29], [30]. Interestingly, these kinases have also been found to be activated during thrombin-induced proliferation of smooth muscle cells under serum-rich conditions [22], . Phosphorylation of p44/p42 MAPK has been linked to subsequent activation of transcription factors [22] and inhibition of the AKT upstream kinase, PI3K, was shown to attenuate this activation [31], [32].

**Figure 4:**
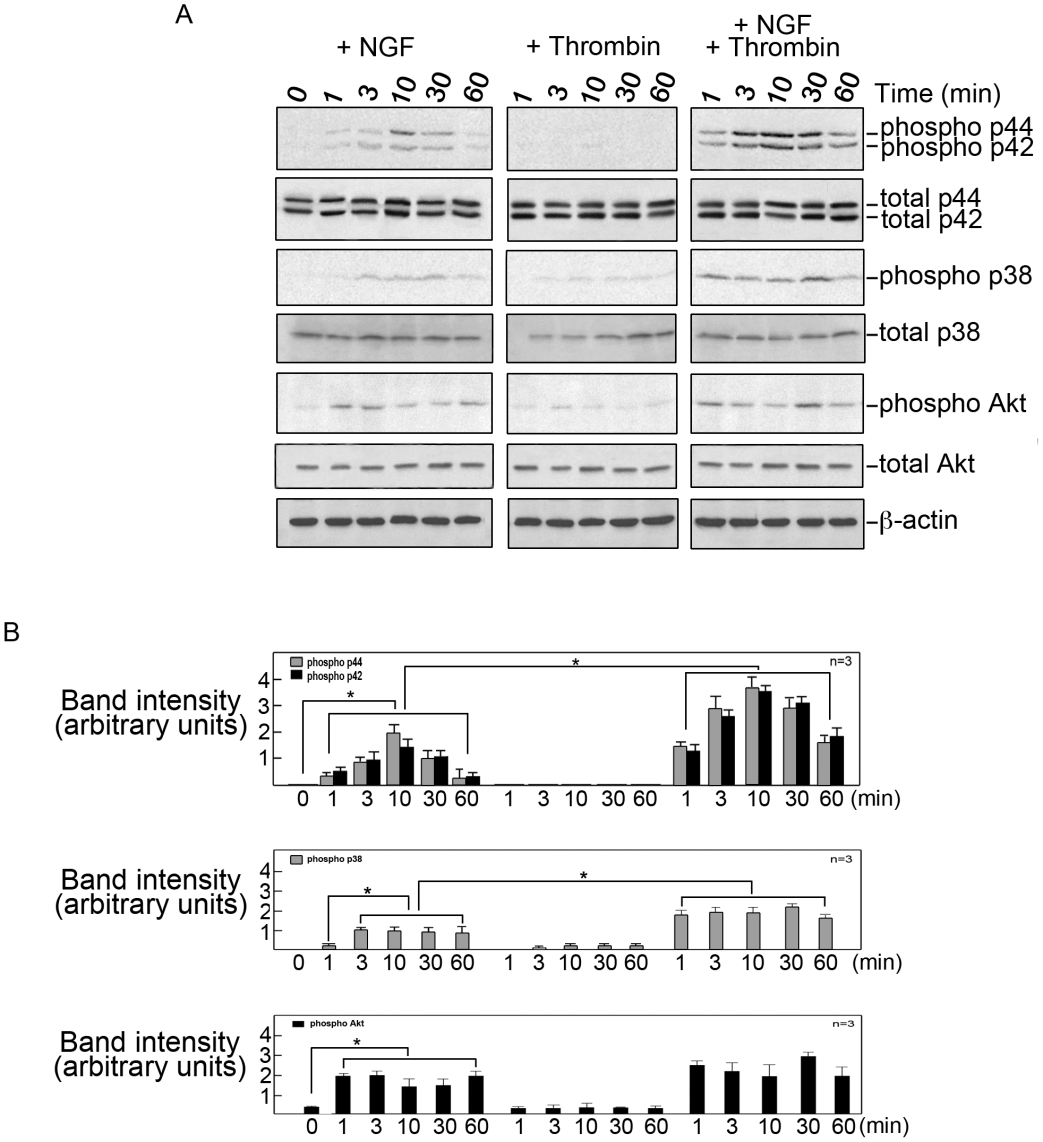
Thrombin enhances NGF-induced neurite outgrowth in differentiating cells via enhanced and sustained activation of p44/42 and p38 MAPK. A. PC12 cells were plated as described in Materials and Methods. After serum starvation for 18 hours, cells were treated with NGF alone (left panels), thrombin alone (middle panels), or NGF+thrombin simultaneously (right panels) under serum-starved conditions, and lysed at 0, 1, 3, 10, 30 and 60 minutes post-treatment. Equal amounts of protein samples were then subjected to western blot analysis using phospho-antibodies against p44/42 MAPK, p38 MAPK and AKT. Immunoblots for total amounts of these proteins were used as internal controls. Equivalent loading was assessed by immunoblotting for β-actin. B. Intensities (in square pixels) of the immunoreactive bands in (A) were measured by densitometric analysis using the NIH Image 1.61 software. Values are means±SD of three separate experiments and indicated by an asterisk (*). Nerve growth factor, NGF.

Therefore, to determine the signaling pathway through which thrombin enhances NGF-induced neurite outgrowth in differentiating cells, serum-starved PC12 cells treated with NGF alone, thrombin alone or thrombin+NGF were lysed at different time points following treatment and examined for p44/42 MAPK, p38 MAPK and AKT activation. Consistent with previous reports [31], [32], we found activation of p44/42 MAPK, p38 MAPK and AKT in NGF-stimulated PC12 cells (Figure 4, A and B, left panels). Treatment with thrombin alone, did not cause significant activation of any of these kinases (Figure 4, A and B, middle panels). However, simultaneous treatment with NGF and thrombin resulted in enhanced and sustained activation of p44/42 MAPK and p38 MAPK (Figure 4, A and B, right panels) although activation of AKT was only modestly enhanced compared to that observed in cells treated with NGF alone. These findings support our premise that thrombin stimulates separate signaling pathways in differentiated and differentiating cells. Thus, thrombin activates AKT to induce retraction of neurites in NGF-differentiated PC12 cells but activates p44/42 MAPK and p38 MAPK to enhance NGF-induced neurite extension in differentiating PC12 cells.

Although the mechanism on how thrombin activates AKT while causing neurite retraction in differentiated PC12 cells remains to be investigated, our finding seems to parallel the observation that AKT activity is not inhibited by lysophosphatidic acid-induced neurite retraction [33]. In addition, while enhanced and sustained activation of p44/42 MAPK have also been associated with apoptosis in primary cortical neurons [34], we did not observe significant PC12 cell death following simultaneous treatment with NGF and thrombin (data not shown). This may be explained by the fact that NGF also exerts a neuroprotective effect on PC12 cells through the PI3K/AKT pathway, which is important for cell survival [6]. Indeed, AKT has been found to be responsible for approximately 80% of survival in NGF-treated cells [2]. In this study, we noted sustained and almost comparable extent of AKT activation in cells treated with NGF alone and in cells treated with NGF+thrombin. Our finding, however, that inhibition of PI3K, an AKT upstream kinase, does not significantly alter the effect of NGF+thrombin further suggests that the PI3K/AKT pathway is not involved in the thrombin enhancement of NGF-induced neurite extension in differentiating PC12 cells.

**Figure 5:**
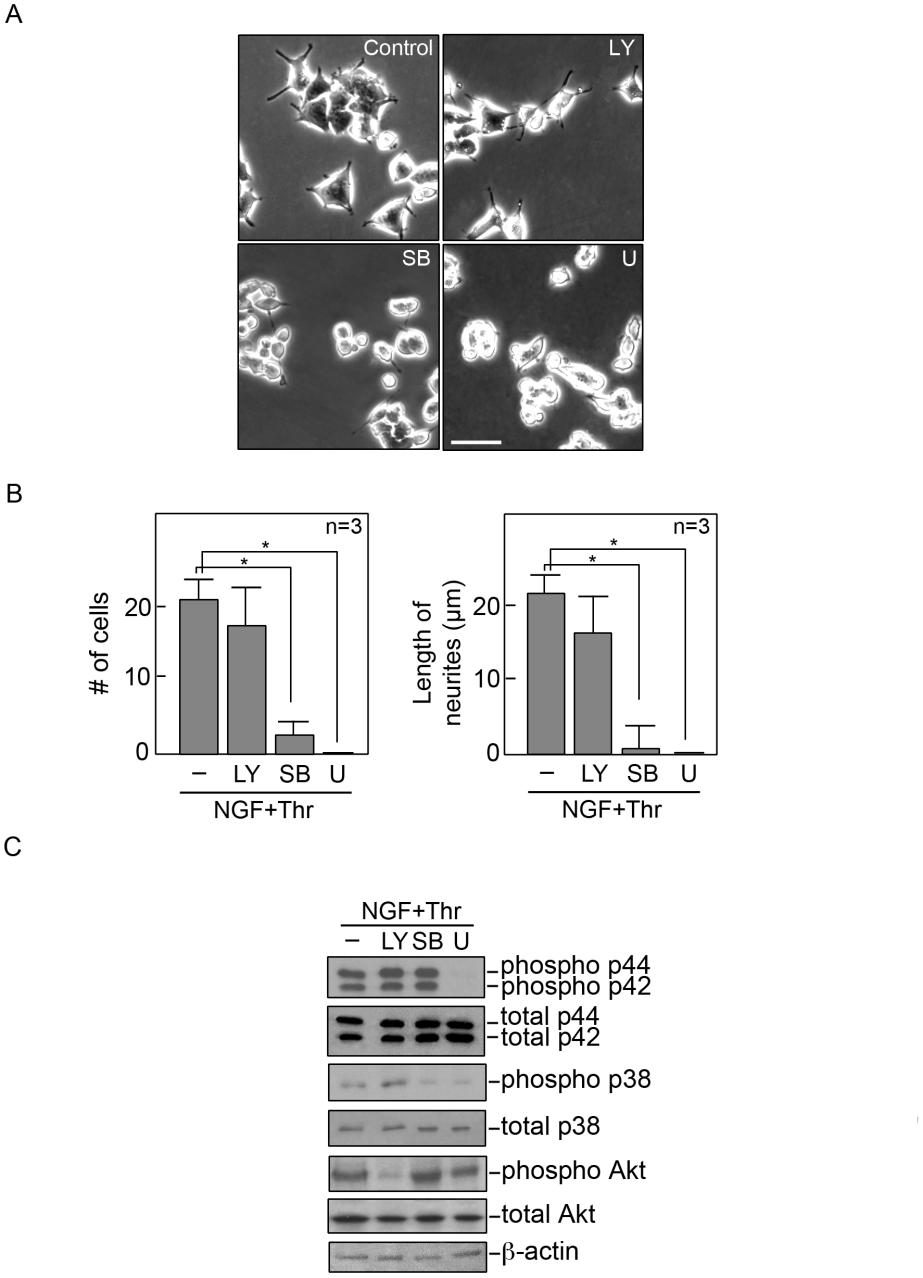
Inhibition of p44/42 and p38 MAPK blocks NGF+thrombin-induced neurite extension in PC12 cells. A. Cells were plated as described in Materials and Methods. After serum starvation for 18 hours, cells were treated with 10 µM of SB203580 (SB; p38 MAPK inhibitor) or U0126 (U; p44/42 MAPK inhibitor), or 30 µM of LY294002 (LY; inhibitor of the AKT upstream kinase, PI3K) for 30 min prior to treatment with NGF+thrombin under serum-starved conditions. Cells were then photographed 48 hours after culture with or without NGF- and thrombin-containing DM. Control, no inhibitor added. Scale bar = 60 µm. The left panel shows the number of cells with neurites longer than 20 µm in each of the 4 treatment groups. The right panel shows the average length of neurites in each of the 4 treatment groups. Measurements were taken from 3 sets of 150 cells for each treatment group. The three sets were from 3 representative experiments. Statistical significance, using one-way ANOVA, was set at p≤0.01 and indicated by an asterisk (*). Nerve growth factor, NGF; thrombin, Thr; -, no inhibitor added. C. Cells were grown as indicated in (A) above. After pre-incubation with LY, SB or U for 30 min, cells were treated with NGF and thrombin for 30 min, lysed and subjected to western blot analysis using phospho-antibodies against p44/42 MAPK, p38 MAPK and AKT. Immunoblots for total amounts of these proteins were used as internal controls. A β-actin blot was used to further assess equivalent loading of samples.

To further investigate the involvement of p44/42 MAPK and p38 MAPK in the thrombin+NGF-induced neurite extension in differentiating cells, we took advantage of the p44/42 MAPK and p38 MAPK inhibitors, U0126 and SB203580, respectively. Serum starved PC12 cells were exposed to SB203580 or U0126 prior to simultaneous treatment with NGF and thrombin. A set of cells was also pre-exposed to LY294002 to verify that AKT does not play an important role in NGF+thrombin-induced neurite extension. After 48 hours in DM containing NGF and thrombin, we observed inhibition of neurite extension in cells treated with SB203580 or U0126 (Figure 5, A and B). Cells exposed to LY294002 showed only a modest decrease in neurite extension. No obvious effects of these inhibitors on cell viability was observed. By western blot analysis, we found that we have effectively inhibited p44/42 MAPK, p38 MAPK and AKT using U0126, SB203580, and LY294002, respectively.

It is important to note that although thrombin alone does not elicit activation of p44/p42 MAPK, p38 MAPK and AKT in cells under serum-starved and NGF-free conditions, we cannot rule out the possibility that thrombin by itself stimulates other cellular events during these conditions. However, these events do not appear to include regulation of neurites and activation of p44/p42 MAPK, p38 MAPK and AKT.

In summary, our studies provide clear indications that thrombin elicits opposing effects on neurites in differentiated and differentiating neurons via activation of distinct signaling pathways. Indeed, thrombin has also been recently shown to utilize diverse signaling to regulate long-term potentiation (LTP) and threshold for synaptic plasticity through PAR-1 [35]. Our finding that thrombin acts in concert with NGF to enhance neurite extension through the p44/42 MAPK and p38 MAPK signaling pathways is novel and may have important implications such as in the development of the nervous system and the diseases associated with it. Potentially, combined thrombin and NGF therapy could effectively improve the symptoms of impaired learning and memory, and/or spinal cord injury.
